# Evaluation of Lower Limb Arteriovenous Diameters in Indoor Soccer Athletes: Arterial Doppler Ultrasound Study

**DOI:** 10.3389/fphys.2021.687613

**Published:** 2021-06-28

**Authors:** Sónia Mateus, Rui Paulo, Patrícia Coelho, Francisco Rodrigues, Vasco Marques, Henrique P. Neiva, Pedro Duarte-Mendes

**Affiliations:** ^1^EPE–Neurovascular and Cardíac Ultrasound Lab, Espiríto Santo of Évora Hospital, Évora, Portugal; ^2^Sport, Health and Exercise Research Unit, Polytechnic Institute of Castelo Branco, Castelo Branco, Portugal; ^3^Department of Clinical Physiology, Polytechnic Institute of Castelo Branco, Castelo Branco, Portugal; ^4^Department of Sports and Well-being, Polytechnic Institute of Castelo Branco, Castelo Branco, Portugal; ^5^Research Unit in Education and Community Intervention, Viseu, Portugal; ^6^Quality of Life in the Rural World, Polytechnic Institute of Castelo Branco, Castelo Branco, Portugal; ^7^Department Biomedical Sciences Laboratory, Polytechnic Institute of Castelo Branco, Castelo Branco, Portugal; ^8^Vascular Ultrasound Laboratory, Angiology and Vascular Surgery Service, Northern Lisbon University Hospital Centre, Lisbon, Portugal; ^9^Department of Sport Sciences, University of Beira Interior, Covilhã, Portugal; ^10^Research Center in Sports Sciences, Health Sciences and Human Development, CIDESD, Covilhã, Portugal

**Keywords:** arteries, athletes, lower limb, ultrasound, veins

## Abstract

The purpose of this study was to analyze the arterial and venous diameters of lower limbs in indoor soccer athletes and non-athletes using Doppler ultrasound to identify the differences in the variation of arterial and venous diameters between groups. Additionally, we intended to verify the differences of arterial and venous diameters between the skilled member (right member) and the not skilled member in each group. 74 male volunteers, aged between 19 and 30 years old, were divided in a group of athletes (*n* = 37, 24 ± 2.7 years, soccer players from national championship), and a group of non-athletes (*n* = 37, 26 ± 2.83 years). Vascular lower limb was assessed using Doppler ultrasound (Philips HD7 echograph with linear transducer 7–12 MHz). The athletes showed higher diameters of right common femoral artery (*p* = 0.009; moderate), left common femoral artery (*p* = 0.005; moderate), right deep femoral artery (*p* = 0.013; moderate), right popliteal artery (*p* = 0.003; moderate), and left popliteal artery (*p* = 0.017; small) than non-athletes. Veins’ diameters were also higher in athletes, specifically the right deep femoral vein (*p* ≤ 0.001; large), left deep femoral vein (*p* ≤ 0.001; large), right popliteal vein (*p* ≤ 0.001; large), and left popliteal vein (*p* ≤ 0.001; large). Differences were found between the skilled and non-skilled leg in athletes in the popliteal vein (7.68 ± 1.44 mm vs. 7.22 ± 1.09 mm, respectively, *p* < 0.003). It seems that futsal athletes have superior mean diameters of lower limbs arteries and veins of the deep venous system to non-athletes. Moreover, the veins presented greater dilation, namely of the leg of the skilled lower limb.

## Introduction

Arterial and venous systems are different systems, namely in their constitution, giving them very specific characteristics, such as plasticity. It is agreed that the peripheral arterial system has arterial remodeling (Thijssen et al., 2011). This remodeling occurs in different ways regarding the structure and arterial function of athletes, initially dilating the vessel and maintaining it later, depending on physical training (Thijssen et al., 2010). According to Brown (2003) the impact of training endurance exercises on the expansion of vessels, namely the capillaries, is well established, with an increase in the size of the arteries that supply the muscles in effort in individuals who practice endurance exercises (Brown, 2003). These increases can be found in the arteries that nourish the active muscles, suggesting that the increase in arterial diameter is associated with the repeated increase in blood pressure caused by the peak blood flow responses to exertion (Sugawara et al., 2007). This process results in the remodeling of the diameter of the vessel that is mediated by the endothelium (Sugawara et al., 2007). Contrarily, while at rest, the diameter may not be increased, due to compensatory increases in vasoconstrictor tone to maintain blood pressure (Sugawara et al., 2007). These changes in the diameter of the vessels cause significant changes in their ability to conduct blood during effort and rest, namely when there is a reduction in turbulent flow passing to a laminar pattern, consequently increasing the conductance of the vessel in proportion to the fourth power of its diameter (Green et al., 2010; Naylor et al., 2011). The vasodilation capacity of vessels during and after exercise allows blood pressure to be maintained, reducing total peripheral resistance and its plasticity in response to physical exercise, and suggests that arterial adaptation is an essential condition for performance in endurance exercise (Calbet et al., 2004; Saltin and Calbet, 2006; Taylor et al., 2008). According to the literature, it is also possible to observe changes in the venous system. Veins are vessels that, compared to arteries, have a thinner middle layer, with fewer muscle fibers, and therefore less plasticity (Cunha da Silva et al., 2010).

It was previously described that some anaerobic exercises with muscle overload might aggravate venous disorders. However, no similar studies focusing on aerobic exercise were found (Cunha da Silva et al., 2010). According to the literature, exercise increases blood return and the veins of the superficial venous system and communicating veins dilate and become more visible. At the level of the deep venous system, the effects are not clear. It is known that this system houses about 80–90% of the circulating blood and the superficial the rest, so the increase in return will also be greater in the deep system, as well as the possible dilation (Rowland, 2001). Adequate physical exercise is considered an effective measure for the prevention and treatment of chronic venous diseases that occur in the superficial venous system. It is also important to understand the repercussions in the deep venous system, where the most frequent pathology is venous thrombosis, which in turn can result in pulmonary embolism and be fatal (Beebe-Dimmer et al., 2005). These adaptations should be further understood in some specific popular exercises, such as team sports. Among these, indoor soccer emerges as an intermittent sport that requires high-intensity short-duration activities of approximately 3 s, such as sprints, changes of direction, dribbles, jumps, shots, tackling, and short periods of recovery (20–30 s) during the game (Marques et al., 2019). It is one of the most physically demanding team sports, taking into account the concentric and eccentric muscular movements of the lower limbs that were a consequence of the specific actions of the game (Nunes et al., 2018; Torres-Torrelo et al., 2018).

In order to qualitatively and quantitatively assess vessel morphology and flow dynamics in the main arteries and veins of the lower limbs, there are complementary diagnostic tests such as peripheral arterial and venous Doppler ultrasound. The latter is a non-invasive, painless examination, which does not involve the use of radiation and has high applicability and reproducibility (Hwang, 2017). The main objective of the present study was to analyze the arterial and venous diameters of the lower limbs in indoor soccer athletes and non-athletes, using arterial and venous lower limbs Doppler ultrasound was used to identify the differences in the variation of arterial and venous diameters between groups. Additionally, we intended to verify the differences of arterial and venous diameters between the skilled member (right member) and the not skilled member in each group (athletes and non-athletes). We believe that we can verify vessels of greater caliber in athletes, as they are subject to greater blood pressure during exercise and observe larger diameters at the venous level because they are vessels with less elastic properties and less capacity for retraction.

## Materials and Methods

### Participants

An analytical cross-sectional study was carried out on a sample consisting of 74 male individuals aged between 19 and 30 years old who were divided into two groups: a group of athletes (*n* = 37) (mean age 24 ± 2.7 years) and a group of non-athletes (*n* = 37) (mean age 26 ± 2.83 years). Only the participants in which the right lower limb was the skilled one were considered for the study. Athletes were considered individuals who practiced indoor soccer three or more times a week playing in the second national league in central Portugal with the same level of practice (16 ± 2.6 years of practice). The sample was collected in two indoor soccer clubs in the central region. In the group of athletes, goalkeepers were excluded. Inclusion criteria were male athletes or non-athletes without musculoskeletal or neurological injuries, conditions, or syndromes diagnosed in the last 6 months who agreed to participate in the study and underwent arterial and venous lower limb Doppler ultrasound. The group of non-athletes answered the Physical Habitual Activity Questionnaire (Rowland, 2001; Beebe-Dimmer et al., 2005), where it was observed that some individuals had practiced different sports for at least 3 years and at a recreational level, including tennis (*n* = 8), football (*n* = 14), basketball (*n* = 4), handball (*n* = 6), and volleyball (*n* = 5). Those who practice less than three times a week and were not federated in any sport were considered non-athletes, consistent with the criteria adopted in previous studies (Hirsch et al., 2006; Gerhard-herman et al., 2017). All individuals were informed about the purpose of the study and signed the informed consent form. This study was conducted in accordance with the Helsinki Declaration (World Medical Association [WMA], 2008), and all procedures were approved by the local Ethics Committee (14/CE-ESALD/2016).

### Instruments

In order to carry out this study, everyone underwent arterial and venous lower limb Doppler ultrasound and, for this, a Philips ultrasound device and a linear probe 7–12 megahertz (MHz) were necessary. Three techniques were applied, namely ultrasound, pulsed Doppler, and color-coded Doppler, which together allowed for the evaluation of the anatomy and morphology of the vessels and to evaluate the flow of blood vessels (arteries or veins) (Hirsch et al., 2006; Hwang, 2017).

An ultrasound device is a device that uses the reflection of an ultrasound beam through an anatomical structure or erythrocytes to make medical images. The probe through piezoelectric crystals emits and receives ultrasounds with different frequencies, depending on several characteristics of the structure under study (Hirsch et al., 2006; Hwang, 2017).

### Procedures

**FIGURE 1:**
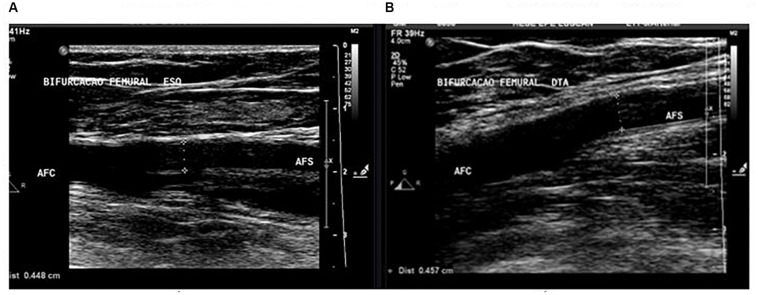
Measurement of arterial diameter of the left **(A)** and right **(B)** superficial femoral artery by lower limb arterial Doppler ultrasound.

Some questions were asked by the researchers, to all individuals, and information about physical activity and the number of times they exercised were recorded. Information on the results of the examinations was also recorded. To perform the exams, it was necessary to have an adequate, quiet physical space, with a low light environment, a tilt table of 30°, an experienced technician, and an ultrasound with a linear probe of 7–12 MHz. The examinations were performed in the supine position and three techniques were applied: ultrasound, pulsed Doppler, and color-coded Doppler. The ultrasound technique allows the visualization of the arterial and venous lumen of the lower limb, allowing an anatomical and morphological analysis, where the diameters of each artery were measured in the longitudinal axis, three times in centimeters (cm), about 2 cm after their origin, with the later calculation using the average of the three measurements (Figures 1A,B).

**FIGURE 2:**
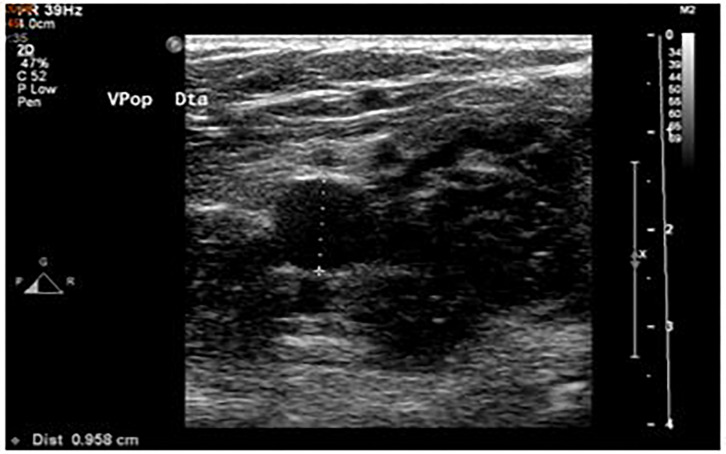
Measurement of venous diameter of right popliteal vein by lower limb venous Doppler ultrasound.

Similarly, for the diameters of the veins, but in transverse axis, we also obtained three measurements, with subsequent calculation of the average, in centimeters (cm): the deep femoral vein 2 cm before the femoral bifurcation and the popliteal vein 2 cm before the saphenous-popliteal cross (Figure 2).

In total, eight arteries and four veins of the deep venous system were studied in all individuals: common femoral arteries (CFA), superficial femoral arteries (SFA), deep femoral arteries (DFA), popliteal arteries (popA), deep femoral veins (DFV), and popliteal veins (popV). The selection of the studied vessels was made to obtain measurements with greater precision and also because they represent the main vessels that carry blood to the thigh muscles. Regarding the veins, the veins of the smaller venous deep system were chosen, but which still allow measurements to be performed with good accuracy representing the thigh and leg venous return, where blood flow during the knee extension exercise is the high point stress during exercise. All artery measurements were made with a longitudinal axis with correct *preset* corrections and all venous measurements were made with a transversal axis with correct veins *preset* corrections. Examinations and results were performed based on the protocols and normality criteria of a study published in 2017 (Hwang, 2017). To assist in conducting the exams, the DGS (2015) protocol on clinical indications and execution methodology was also used (George, 2015).

### Statistical Analysis

#### Preliminary Analysis

An inspection of the data revealed no missing values, nor were univariate outliers found. *A priori* power analysis through G^∗^Power (3.1.9.2) (Faul et al., 2007) was used to determine the required sample size considering the following input parameters: (effect size *d* = 0.8; α = 0.05; statistical power = 0.90). The required sample size was 68 (34 for each group) which was respected in the present study.

#### Main Statistical Analysis

Descriptive statistics including mean and standard deviation were performed for all variables under analysis. The coefficient of variation (CoV) and the interclass correlation coefficient (ICC) was computed to evaluate the variability of the data. Then, a Shapiro–Wilk test (*n* < 50) to analyze data distribution was performed, considering *p* > 0.05 as a normal distribution (Hair et al., 2019). Initially, a *T*-Test for Independent Samples (variables with normal distribution) and Mann–Whitney (variables with non-normal distribution) was used to verify differences between groups (athletes and non-athletes). Secondly, the Paired Sample *T*-test (variables with normal distribution) and Wilcoxon (variables with non-normal distribution) were used to verify the differences of arterial and venues diameters between the skilled member (right member) and the non-skilled member in each group (athletes and non-athletes) (Hopkins et al., 2009). Finally, an effect size (Cohen d) analysis was used to determine the magnitude of effect and the following cut-off values were considered: 0–0.2, trivial; 0.21–0.6, small; 0.61–1.2, moderate; 1.21–2.0, big; and >2.0, very big (George, 2015). In the variables with non-normal distribution, the effect size was calculated based on the eta square value (η^2^) (Lenhard and Lenhard, 2016). All statistical analysis was performed using SPSS software v. 25.0 (IBM, Chicago, IL, United States), and the significance level was set at *p* ≤ 0.05 to reject the null hypothesis (Ho, 2014; Hair et al., 2019).

## Results

Table 1 shows the differences in the studied variables between the two groups (athletes and non-athletes) on the diameter of the right and left arteries and veins. Differences between groups (*p* ≤ 0.05) in the diameter of right common femoral artery (*p* = 0.009; moderate), in the diameter of left common femoral artery (*p* = 0.005; moderate), in the diameter of right deep femoral artery (*p* = 0.013; moderate), in the diameter of right popliteal artery (*p* = 0.003; moderate), and in the diameter of left popliteal artery (*p* = 0.017; small) were found. We also found differences in the diameter of the right deep femoral vein (*p* ≤ 0.001; big), in the diameter of the left deep femoral vein (*p* ≤ 0.001; big), in the diameter of the right popliteal vein (*p* ≤ 0.001; big), and in the diameter of the left popliteal vein (*p* ≤ 0.001; big), showing that the group of athletes had the highest values.

**TABLE 1:** Descriptive statistics, differences between groups, and effect size in the variation of diameter of arteries and veins of left and right limbs.

Variables	Groups*N*	M	M CI 95%	SD	*p*	Effect size	ICC (CI 95%)	CoV
Diameter – right common femoral artery	Non-athletes37	7.52	7.18–7.86	1.01	0.009^a^*	0.621	0.998 (0.996–0.999)	1.2%
	Athletes37	8.14	7.81–8.47	0.99			0.993 (0.988–0.996)	2.1%
Diameter – left common femoral artery	Non-athletes37	7.41	7.12–7.68	0.85	0.005^a^*	0.663	0.997 (0.994–0.998)	1.3%
	Athletes37	7.97	7.69–8.25	0.84			0.983 (0.971–0.991)	2.8%
Diameter – right deep femoral artery	Non-athletes37	5.19	4.99–5.40	0.61	0.013^a^*	0.605	0.966 (0.942–0.982)	3.1%
	Athletes37	5.68	5.35–6.00	0.97			0.994 (0.99–0.997)	3.2%
Diameter – left deep femoral artery	Non-athletes37	5.23	4.98–5.48	0.75	0.581^b^	0.451	0.99 (0.982–0.994)	2.5%
	Athletes37	5.62	5.34–5.89	0.82			0.96 (0.931–0.978)	3.8%
Diameter – right superficial femoral artery	Non-athletes37	6.24	6.02–6.45	0.65	0.197^a^	0.292	0.988 (0.979–0.993)	2.3%
	Athletes37	6.40	6.26–6.55	0.43			0.961 (0.933–0.979	3.1%
Diameter – left superficial femoral artery	Non-athletes37	6.40	6.13–6.67	0.80	0.228^a^	0.227	0.989 (0.981–0.994)	2.6%
	Athletes37	6.59	6.41–6.78	0.55			0.966 (0.942–0.981)	3.6%
Diameter – right popliteal artery	Non-athletes37	5.69	5.46–5.93	0.72	0.003^a^*	0.734	0.989 (0.981–0.994)	2.9%
	Athletes37	6.12	5.99–6.26	0.41			0.95 (0.913–0.972)	3.7%
Diameter – left popliteal artery	Non-athletes37	5.84	5.60–6.07	0.70	0.017^a^*	0.568	0.985 (0.975–0.992)	3.0%
	Athletes37	6.16	6.03–6.28	0.38			0.952 (0.918–0.974)	3.5%
Diameter – right deep femoral vein	Non-athletes37	6.75	6.11–7.39	1.91	<0.001^a^*	1.323	0.997 (0.996–0.999)	2.7%
	Athletes37	9.31	8.65–9.96	1.96			0.998 (0.996–0.999)	2.7%
Diameter – left deep femoral vein	Non-athletes37	6.39	5.69–7.08	2.09	<0.001^b^*	1.692	0.999 (0.998–0.999)	2.4%
	Athletes37	9.68	9.09–10.28	1.79			0.998 (0.997–0.999)	2.2%
Diameter – right popliteal vein	Non-athletes37	5.32	4.76–5.88	1.68	<0.001^a^*	1.508	0.998 (0.996–0.999)	3.0%
	Athletes37	7.68	7.20–8.15	1.44			0.997 (0.994–0.998)	2.5%
Diameter – left popliteal vein	Non-athletes37	5.32	4.88–5.77	1.33	<0.001^a^*	1.563	0.997 (0.995–0.998)	2.9%
	Athletes37	7.22	6.86–7.58	1.09			0.983 (0.971–0.991)	4.6%

*p ≤ 0.05 – a student’s *t*-test and b Mann–Whitney *U* test significance level; *p* > 0.05 – Levene’s test equal variances assumed. N, subjects’ number; M, mean; M CI, Confidence interval for the mean values; SD, standard deviation; CI, confidence interval; diameter – mm; ICC, intra-class correlation coefficient; CoV, coefficient of variation.

**FIGURE 3:**
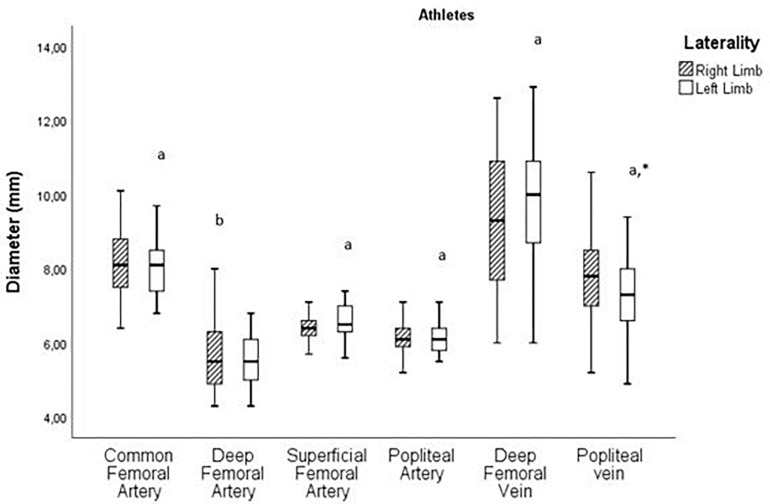
Analysis of the diameter variance pairwise of right and left arteries and veins in athletes (*p ≤ 0.05; ^a^ Paired *T*-Test; ^b^ Wilcoxon).

Figure 3 shows the variance analyses related to the diameter of the right and left arteries and veins in the athletes. Statistically acceptable values (*p* ≤ 0.05) were found in popliteal vein.

**FIGURE 4:**
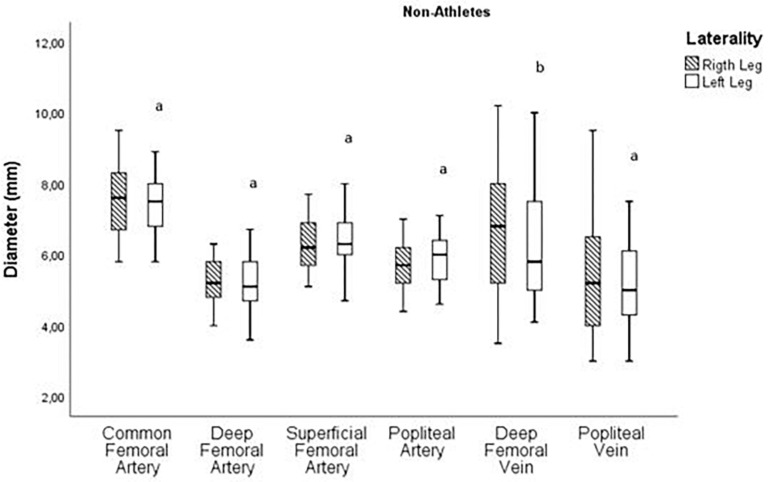
Analysis of the diameter variance pairwise of right and left arteries and veins in non-athletes (^a^ Paired *T*-Test; ^b^ Wilcoxon).

Figure 4 shows the variance analyses related to the diameter of the right and left arteries and veins in the non-athletes. Statistically acceptable values were not found.

## Discussion

The main objective of the present study was to analyze the arterial and venous diameters of the lower limbs in indoor soccer athletes, who play an intermittent high-intensity team sport, and non-athletes (Ramos-Campo et al., 2016), using arterial and venous lower limb Doppler ultrasound to identify the differences in the variation of the arterial and venous diameters between groups. Additionally, we intended to verify the differences in the arterial and venous diameters between the skilled member (right member) and the not skilled member in each group (athletes and non-athletes). Like other studies, Doppler ultrasound was the diagnostic test selected due to its high sensitivity in arterial and venous studies. It is the gold standard diagnostic exam in venous evaluation. The Doppler ultrasound is a technique that allows the anatomical and morphological evaluation of vessels and hemodynamic study of blood qualitatively and quantitatively, being non-invasive and painless for the patient (Hussain et al., 1996; Thrush and Hartshorne, 2005).

Previous studies have reported increases in the diameter of the arteries that supply the muscles during endurance exercises (Thijssen et al., 2010). Nevertheless, they have not reported a study of this variation in diameters between lower limb arteries in indoor soccer athletes. Regarding the venous system, only studies were found regarding anaerobic exercises with muscle overload (Cunha da Silva et al., 2010).

In the current study, the average diameter of the arteries and veins studied in athletes was higher than non-athletes in both limbs. These differences were statistically significant in almost all studied arteries and all studied veins. At the same time, the minimum values of arterial and venous diameters are also always higher in the group of athletes. We think that a possible explanation of these findings is the specific demands of indoor soccer that involves high-intensity short duration activities that require very high concentric and eccentric muscular movements in the lower limbs (Marques et al., 2019). These results corroborate with previous studies that show that athletes’ arteries have larger lumen diameters than healthy non-athletes’ arteries (Green et al., 2012). Also, two studies from Tinken et al. (2008, 2010) stated that athletes’ arteries may be normal in function, but different in some structural characteristics. These characteristics can happen through changes in arterial structure and function, where dilation occurs initially, which, depending on the intensity of the training, will be maintained later. According to the last study, there is sufficient evidence to conclude that endurance athletes have enlarged arteries and may also have decreased wall thickness (Tinken et al., 2010). In this sample, the fact that both SFA do not present statistically significant differences may be related to the artery having a greater length and a greater number of branches of the thigh, with these anatomical characteristics allowing for a better distribution and dispersion of the increased blood gradient during exercise, creating less impact on the arterial vascular wall, and thereby decreasing dilation (Hwang, 2017). Regarding the studied veins, the results are in agreement with the fact that the middle layer of the venous wall has less muscle and elastic fibers, decreasing its plasticity, with the blood pressure being very low compared to the arterial system in the veins. According to literature, the athletes’ venous system during training is subject to an increase in blood return and, consequently, pressure, which may dilate the veins of the superficial venous system too much, which, being connected to the deep venous system by perforating veins, will suffer equal pressure increases and consequent expansion (Cunha da Silva et al., 2010).

In the study of the comparison of arteries between left and right lower limbs, it is observed that in both groups the diameters are higher in the left lower limb, considered the support leg, except for the mean diameter of the CFA which in both groups is higher in the right lower limb, which may be related to this being the skillful leg and anatomically being the artery of shorter length, with a lower number of branches, greater blood volume, and, consequently, an increase in diameter (Hwang, 2017). However, these results were not statistically significant.

As for the comparison in the veins between lower limbs, in the group of non-athletes, the average diameter of the studied veins tends to be higher in the right lower limb, the result being significant in PFVs, veins that reflect the venous return at the level of the thigh muscles of individuals who, despite not being athletes, practice some physical exercise, namely exercises with different characteristics in terms of muscular effort. In the athletes’ group, the mean diameter of the studied veins is higher in the left lower limb at the level of PFV, with the opposite occurring at the level of the popV, which has mean diameters greater in the right, which is a significant result. This may again be related to the skillful leg and greater pressure spikes and to training being more directed at strengthening leg muscle groups (Cunha da Silva et al., 2010).

In the analysis of arteries by members, in the group of non-athletes, the variation between the different arteries is significant in the right and left lower limbs. However, in the athletes’ group, the variation between the different arteries is not statistically significant between SFA and popA bilaterally, which can be justified because they are anatomically similar in caliber to the others studied (Hwang, 2017).

In the analysis of the venous system by members, in the non-athletes’ group, there was statistical significance in the variation between the means of the venous diameters on the left and in the right. In the athletes’ group, statistical significance was only found in the right. This means that there are differences in blood pressure between the members of futsal athletes. This can be justified by the right limb in this sample being the skilled member, with the left leg being the supporting leg. Remembering that the veins have less plasticity, the veins of the skilled member are subject to greater peak pressure and pressure variations, dilate more and stay dilated, not returning to the initial diameter. The veins of the support member suffer fewer pressure peaks and are likely to experience smaller increases in diameter (Cunha da Silva et al., 2010). The results of this study, given the fragility of the vein walls and the possible irreversibility of the dilation they suffer during training, point to the need for a supervised training program and the application of preventive measures. There is some evidence that the practice of exercise is related to the worsening of venous disorders (Cunha da Silva et al., 2010). According to a study published in 2006, venous diameters are strongly related to chronic insufficiency of the veins of the peripheral venous system (Eifell et al., 2006). The dilation of a blood vessel can cause venous reflux and, consequently, an increase in local venous pressure, especially in the lower limbs where, due to the action of the force of gravity, greater pumping is needed for its upward path to the right atrium (Padberg et al., 2004). However, physical exercise can promote benefits in the functionality of the venous system. It is not related to the type of exercise, but to the amount and intensity of training. Aerobic exercise is recommended by the literature for individuals with venous diseases, since it is able to improve the venous pressure drop mechanism and increase venous flow (Kahn et al., 2003; Padberg et al., 2004). During exercise, muscle contraction provides perfusion pressure, helping blood flow from the lower limbs to the heart, opposing forces against venous return such as gravity (Kahn et al., 2003; Padberg et al., 2004). A study where 100 adults over 50 years of age, of both sexes, were prospectively studied to verify the relationship between lower limbs chronic venous insufficiency and physical activity concluded that exercise did not influence the occurrence of venous insufficiency, having prevented the evolution to more advanced stages (Tinken et al., 2010). However, the type of exercise was not clarified, and it was a study applied to individuals older than the present study. The scarce literature on the effect of aerobic and anaerobic exercise on the arterial system, and especially on the venous system, is a restriction that remains.

Although the present study contributes to knowing how sports practice influences the caliber of arteries and veins, it has some limitations. One of the limitations was the comparison of one single sport modality (futsal) against multiple heterogeneous sports activities and the fact that the cofound variables were not analyzed (e.g., hydration, diet, and previous physical activities) (Duarte-Mendes et al., 2020). Another limitation is the fact that objective measures were not used to directly assess one or more dimensions of physical activity (e.g., frequency, intensity, time, or type) (Alberti et al., 2010), and all variables were assessed at one moment (cross-sectional design). Therefore, we cannot draw causality associations. Longitudinal and/or experimental studies are needed to further examine the effects of the analyzed variables. In order to increase knowledge on the variability of lower limb artery and vein diameters in athletes, we suggest future studies considering the wide range of sport modalities and physical activity. As for the practical implications, coaches and players should take into account the observed changes. To minimize them, vasoconstrictor techniques can be applied, such as the use of compression stockings or the application of ice in high-intensity training (Armstrong et al., 2015).

## Conclusion

In this study, the group of futsal athletes had superior mean diameters of the arteries and veins of the deep venous system of the lower limbs to non-athletes. Nevertheless, it was concluded that in the veins that there was greater dilation, namely of the leg of the skilled lower limb. This variation in the diameter of veins in the venous system should be monitored to prevent the functional impact that may lead to chronic venous diseases, with medical monitoring and convenient prevention measures.

## Data Availability Statement

The raw data supporting the conclusions of this article will be made available by the authors, without undue reservation.

## Ethics Statement

The studies involving human participants were reviewed and approved by the Comissão de Ética Escola Superior de Saúde Dr. Lopes Dias. The patients/participants provided their written informed consent to participate in this study.

## Author Contributions

SM, RP, PC, FR, VM, HN, and PD-M participated in study design, data collection, and the writing of the first draft manuscript. SM, PC, FR, and PD-M participated in article collection and analysis. SM, RP, PC, FR, HN, and PD-M participated in the writing of the methodology and results. SM, RP, HN, and PD-M participated in final revisions of the manuscript. All authors have read and approved the final version of the manuscript and agreed with the order of presentation of the authors.

## Conflict of Interest

The authors declare that the research was conducted in the absence of any commercial or financial relationships that could be construed as a potential conflict of interest.
